# Improving radiation dosimetry with an automated micronucleus scoring system: correction of automated scoring errors

**DOI:** 10.1007/s00411-023-01030-7

**Published:** 2023-05-17

**Authors:** Younghyun Lee, Young Woo Jin, Ki Moon Seong, Ruth C. Wilkins, Seongjae Jang

**Affiliations:** 1 Laboratory of Biological Dosimetry, Korea Institute of Radiological and Medical Sciences, National Radiation Emergency Medical Center, Seoul, Republic of Korea; 2 Korea Institute of Radiological and Medical Sciences, National Radiation Emergency Medical Center, Seoul, Republic of Korea; 3 Consumer and Clinical Radiation Protection Bureau, Health Canada, Ottawa, ON Canada; 4 Department of Biomedical Laboratory Science, College of Medical Sciences, Soonchunhyang University, Asan, Republic of Korea

**Keywords:** Biodosimetry, Micronuclei assay, Automated scoring, Radiation exposure

## Abstract

Radiation dose estimations performed by automated counting of micronuclei (MN) have been studied for their utility for triage following large-scale radiological incidents; although speed is essential, it also is essential to estimate radiation doses as accurately as possible for long-term epidemiological follow-up. Our goal in this study was to evaluate and improve the performance of automated MN counting for biodosimetry using the cytokinesis-block micronucleus (CBMN) assay. We measured false detection rates and used them to improve the accuracy of dosimetry. The average false-positive rate for binucleated cells was 1.14%; average false-positive and -negative MN rates were 1.03% and 3.50%, respectively. Detection errors seemed to be correlated with radiation dose. Correction of errors by visual inspection of images used for automated counting, called the semi-automated and manual scoring method, increased accuracy of dose estimation. Our findings suggest that dose assessment of the automated MN scoring system can be improved by subsequent error correction, which could be useful for performing biodosimetry on large numbers of people rapidly, accurately, and efficiently.

**Supplementary Information:**

The online version contains supplementary material available at 10.1007/s00411-023-01030-7.

## Introduction

Following a radiological accident, it is necessary to rapidly perform radiation dosimetry on victims, which will identify those who have suffered overexposure and require urgent medical treatment. In general, the dicentric chromosome assay (DCA) is considered to be the gold standard for such biodosimetry. It has been widely used to evaluate radiation doses of accidentally and occupationally exposed persons (Slozina et al. 2001; Ramalho and Nascimento 1991; Suto et al. 2013; Chung et al. 1996), but it might be not suitable for larger- scale radiological accidents due to multiple drawbacks: it is labor-intensive, time-consuming, and requires highly skilled personnel.

The development of automated systems using alternative tools should be considered to overcome these limitations and increase dosimetry throughput. As counting of micronuclei (MN) is much simpler and faster than the DCA, it has been considered as an alternative. MN are produced by lagging acentric chromosome fragments or whole chromosomes at anaphase (IAEA 2011; Lue et al. 2015). The cytokinesis-block micronucleus (CBMN) assay developed by Morley and Fenech (Fenech and Morley 1985), is a well-established method that exploits this phenomenon for genotoxicity testing. It has been recommended by the Organisation for Economic Co-operation and Development (OECD) for in vitro genotoxicity testing (OECD 2016). It has been reported that MN frequencies in binucleated (BN) cells are strongly correlated with radiation dose (Vral et al. 2011, 1994); the CBMN assay has been recommended as a valuable technique to measure chromosomal damage for biodosimetry (IAEA 2011). The International Organization for Standardization (ISO) has published a guideline on CBMN performance criteria for biodosimetry (ISO 2014).

The simplicity of MN scoring and the availability of automated scoring system through computerized imaging makes the CBMN assay more attractive, especially for large-scale radiological accidents (Depuydt et al. 2017). Multiple attempts have been made to score MN frequencies automatically, using computerized imaging or flow cytometry (Shibai-Ogata et al. 2011). One of these, the MNScore module, is an automated MN scoring system integral to the MetaSystems Metafer 4 image-analysis platform, which is commonly used to find metaphase cells in clinical cytogenetics laboratories. Automation of the CBMN assay with the MNScore module has been introduced as a biodosimetry tool for population triage, but its accuracy relative to manual scoring has not been extensively studied.

From a clinical viewpoint, dosimetry to identify subjects who require urgent clinical needs may provide sufficient information, but it would be desirable to improve accuracy as much as possible to improve long-term epidemiological follow-up (Romm et al. 2013; Rothkamm et al. 2013). Here, we investigated the impacts of automated scoring errors and sex on MN dose–response curves.

## Materials and methods

### Blood samples and irradiation

This study was approved by Institutional Review Board (IRB) of the Korea Institute of Radiological and Medical Sciences (IRB No. K-1707–001-003). Heparinized blood samples were collected from healthy donors (3 males and 3 females with ages ranging from 29 and 34) who provided informed written consent. For dose–response curves, blood samples were irradiated with different doses (0- 4 Gy) of ^60^Co gamma rays at 0.5 Gy/min in a water phantom at 37 ℃. After irradiation, samples were incubated at 37 ℃ for 2 h, then processed for the CBMN assay.

### CBMN assay

Whole-blood samples (1.5 ml) were cultured in 9 ml Roswell Park Memorial Institute (RPMI) 1640 medium (Gibco, Waltham, MA) supplemented with 20% fetal bovine serum (JR Scientific, Woodland, CA), 1% antibiotic–antimycotic (Gibco), and 2% phytohemagglutinin (Gibco) at 37 ℃ and 5% CO_2_ in air. After 24 h of culture, cytochalasin B (Sigma, St. Louis, MO) was added to the cultures at a final concentration of 6 μg/ml. After an additional 48 h of culture, cells were harvested and resuspended in ice-cold hypotonic solution (0.075 M KCl). Cells were fixed once with methanol/acetic acid (10:1) diluted 1:1 with Ringer’s solution, and fixed three more times with methanol/acetic acid without Ringer’s solution. Fixed cells were dropped on slides. To obtain enough BN cells, 1–4 slides per dose point of each donor were made and stained with DAPI (Cytocell, Cambridge, UK).

### MN scoring

DAPI-stained slides were scanned with Metafer 4 software (MetaSystems, Altlussheim, Germany) with 10 × objective. For fully-automated scoring mode, scoring MN in BN cells was performed in MNScore module in Metafer 4 image analysis platform. After automated scoring, images captured with MNScore were reanalyzed by a trained human scorer according to published scoring criteria (Fenech et al. 2003); for semi-automated scoring mode, BN cells with MNScore-detected MN were inspected to eliminate false-positive MN; for manual scoring mode, all BN cells, both with and without detected MN, were completely scored to remove false-positives and false-negatives. False positive BN cells were rejected in semi-automated and manual scoring mode.

### Validation using blind samples

X-irradiated samples (*n* = 10) for dose estimation tests were provided from Health Canada as part of intercomparison exercises for radiation biodosimetry, which was approved by the IRB of Health Canada (approval REB 2002–0012). Blood samples were obtained from 10 donors (6 males, 4 females, age 21–55) after obtaining informed consent. Samples were irradiated with different doses (0, 0.4, 0.8, 1.0, 1.4, 2.0, 2.2, 2.6, 3.2 and 3.6 Gy) at 0.37 Gy/min using an X-RAD 320 device operated at 250 kVp and 15 mA. After irradiation, blood samples were incubated at 37 ℃ for 2 h, coded to blind us to sources, and shipped to our laboratory in the Korea Institute of Radiological and Medical Sciences (KIRAMS). γ-irradiated samples for validation were prepared in KIRAMS, Republic of Korea. For γ-irradiated samples (*n* = 12), blood samples collected from 3 donors (1 male and 2 females, age 35–50) were irradiated with different doses (0, 0.5, 1, 3 Gy) of ^60^Co gamma rays at 0.5 Gy/min in a water phantom at 37 ℃ using GammaBeam 100–80 (Best Theratronics) of KIRAMS. All samples for validation were coded and the CBMN assay was performed as described above.

### Dose estimation and statistical analysis

Fitting of dose–response curves to data from blind samples was performed using Dose Estimate software ver. 5.2, kindly provided from Dr. E.A. Ainsbury of UK Health Security Agency (Ainsbury and Lloyd 2010). The curves for MN were fitted to the linear quadratic model: $y=c+\alpha D +\beta {D}^{2}$, where y is the MN frequency per BN cell, c is the spontaneous MN frequency, α is a linear component of a curve, β is a quadratic component of a curve, and D is the radiation dose. Doses given to the 10 validation samples were estimated with the Dose Estimate software. The 95% upper and lower confidence limits were calculated taking into account Poisson and calibration curve errors (IAEA 2011). To test the discriminatory power (≤ 1.5 Gy/ > 1.5 Gy) of our CBMN assay, sensitivity, specificity and accuracy was calculated according to Rothkamm et al. (2013). We considered the dose estimates to be accurate when their 95% confidence intervals encompassed the known, actual dose.

## Results

### Dose–response calibration curve

The data for micronucleus formation by ^60^Co γ-irradiation obtained from 6 healthy donors (3 males and 3 females) were pooled to construct a dose–response calibration curve (Table 1, Supplementary Tables 1 and 2). Dose response curves were constructed on the average values of 3 males and 3 females. For automated dose response curves, MNScore software in Metafer4 platform scored at least 16,000 binucleated (BN) cells for each dose point.

**Table 1:** Micronucleus frequencies in and distributions lymphocytes from 6 donors (3 males and 3 females pooled) scored by fully-automated method

Dose (Gy)	No. of BN	No. of MN	Distribution of MN					Dispersion index (${\sigma }^{2}/y)$	MN frequency
			0	1	2	3	4		
0	16,020	335	15,706	295	17	2	0	1.12	0.021
0.1	18,000	334	17,676	314	10	0	0	1.04	0.019
0.25	18,000	419	17,602	382	13	1	2	1.11	0.023
0.5	18,000	591	17,440	531	28	0	1	1.08	0.033
0.75	18,000	650	17,371	610	17	2	0	1.03	0.036
1.0	18,000	866	17,167	802	29	2	0	1.03	0.048
2.0	18,000	1861	16,256	1630	111	3	0	1.03	0.103
3.0	17,572	2898	14,933	2400	220	18	1	1.03	0.165
4.0	18,000	4231	14,295	3215	455	34	1	1.03	0.235

*MN* micronucleus, *BN* binucleated cell

To evaluate the accuracy of our automated scoring system, images gallery captured with MNScore were manually inspected. Table 2 shows the false detection rates of BN cells and MN in automated scoring system. After visual inspection, 0.72–2.20% of the auto-selected BN cells were rejected because they did not comply with the standardized scoring criteria (Fenech et al. 2003). Average false-positive and false-negative MN frequencies in the total scored BN cells were 1.03% (range: 0.72–1.50) and 3.50% (range: 1.02–10.78), respectively. The rejected BN cells and false detected MN in automated scoring system seemed to be increased with radiation dose.

**Table 2:** False detection rate of automated micronucleus scoring shown in Table 1

Dose (Gy)	False positive BN frequency (%)^1^	False positive MN frequency (%)^2^	False negative MN frequency (%)^3^
Total	1.14	1.03	3.50
0	0.98	0.67	1.12
0.1	0.72	0.73	1.02
0.25	0.80	0.72	1.33
0.5	1.16	1.02	1.77
0.75	1.09	0.76	1.68
1.0	1.02	1.04	2.04
2.0	0.87	1.36	4.43
3.0	1.38	1.50	7.37
4.0	2.20	1.46	10.78

^1^False positive BN frequency (%) = No. of false positive binucleated (BN) cells / No. of total scored BN cells × 100

^2^False positive MN frequency (%) = No. of false positive micronuclei / No. of total scored BN cells × 100

^3^False negative BN frequency (%) = No. of false negative micronuclei / No. of total scored BN cells × 100

*MN* micronucleus, *BN* binucleated cell

**Fig. 1:**
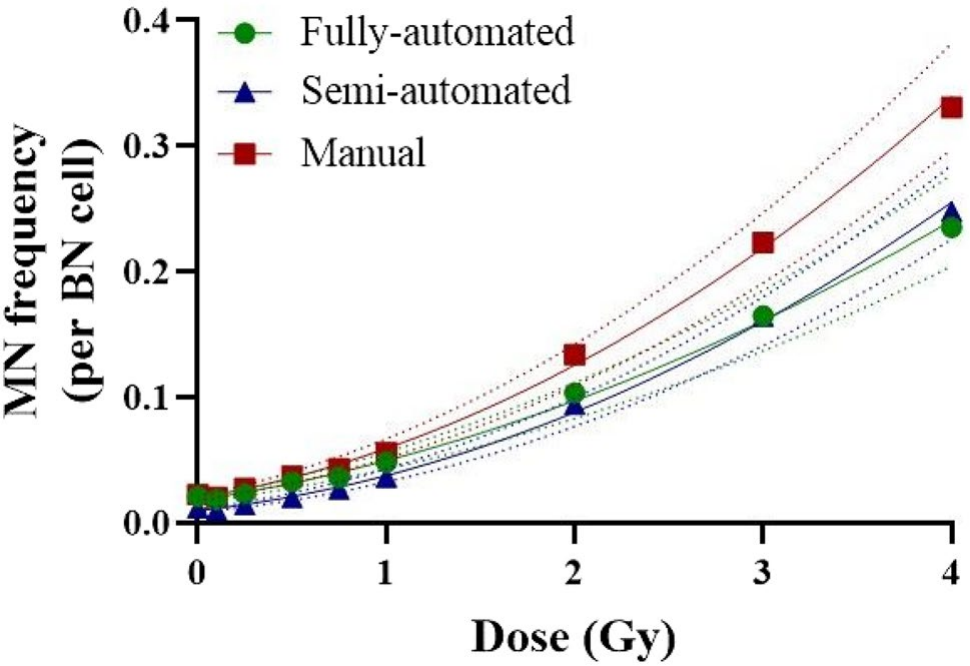
Dose–response curves from micronucleus (MN) data produced by fully-automated, semi-automated and manual scoring. MN yields were fitted to a linear quadratic model: $\mathrm{y}=0.0178 \left(\pm 0.0016\right)+0.0237 \left(\pm 0.0039\right)\times D +0.0080 \left(\pm 0.0012\right)\times {D}^{2}$ in fully-automated scoring method, $\mathrm{y}=0.0096 \left(\pm 0.0011\right)+0.0170 \left(\pm 0.0031\right)\times D +0.0111 \left(\pm 0.0010\right)\times {D}^{2}$ in semi-automated scoring method, $\mathrm{y}=0.0197\left(\pm 0.0018\right)+0.0259 \left(\pm 0.0045\right)\times D +0.0135 \left(\pm 0.0014\right)\times {D}^{2}$ in manual scoring method. Symbols and lines represent the average MN frequencies for 6 subjects and fitted curves. BN: binucleated

Dose–response curves of micronuclei described in Fig. 1 were fitted using a linear quadratic equation using DoseEstimate v5.2. The equations regenerated as: $\mathrm{y}=0.0178 \left(\pm 0.0016\right)+0.0237 \left(\pm 0.0039\right)\times D +0.0080 \left(\pm 0.0012\right)\times {D}^{2}$ in the fully automated scoring method, $\mathrm{y}=0.0096 \left(\pm 0.0011\right)+0.0170 \left(\pm 0.0031\right)\times D +0.0111 \left(\pm 0.0010\right)\times {D}^{2}$ in the semi-automated scoring method and $\mathrm{y}=0.0197\left(\pm 0.0018\right)+0.0259 \left(\pm 0.0045\right)\times D +0.0135 \left(\pm 0.0014\right)\times {D}^{2}$ in the manual scoring method.

### Radiation dose prediction

For the dose prediction exercise, we estimated the radiation dose of 22 blind samples irradiated with different doses of X-rays or γ-rays by calculating the MN frequency observed with fully-automated, semi-automated and manual modes (Supplementary Tables 3 and 4). To test the performance of our automated scoring system for triage in a large-scale radiological incident, we merged dose measurements into binary categories reflecting clinically relevant aspects. The sensitivity, specificity and accuracy based on MN measurements using automated, semi-automated and manual modes are summarized in Table 3. The sensitivity, specificity and accuracy to detect MN and non-MN correctly in total BN cells was 1.0, 0.20, and 0.56 in the fully-automated mode, respectively. Our automated scoring system with high sensitivity seemed to be sufficient to identify subjects who are likely to suffer from acute radiation syndrome several days after radiation exposure, but the ability to define persons exposed to below 1.5 Gy from higher exposed group was low. Visual inspection after automated scoring overcame the poor specificity of fully-automated scoring. The sensitivity, specificity and accuracy in the semi-automated and manual mode was 1.0, 0.90 and 0.94, respectively. When splitting data according to radiation source, similar results were observed and γ-irradiated samples have particularly higher specificity and accuracy than X-irradiated ones. These data show that additional visual inspection improves the performance of automated scoring to better identify subjects who need less urgent clinical attention.

**Table 3:** Sensitivity, specificity and accuracy of triage classification in the automated micronucleus (MN) assay

	Estimated dose (Gy)					
	Fully-automated		Semi-automated		Manual	
	≤ 1.5	> 1.5	≤ 1.5	> 1.5	≤ 1.5	> 1.5
All						
Delivered dose (Gy)						
≤ 1.5^1^	2	8	9	1	9	1
> 1.5	0	8	0	8	0	8
Sensitivity^2^	1.00		1.00		1.00	
Specificity^3^	0.20		0.90		0.90	
Accuracy^4^	0.56		0.94		0.94	
X-irradiated						
Delivered dose (Gy)						
≤ 1.5^1^	1	3	3	1	3	1
> 1.5	0	5	0	5	0	5
Sensitivity^2^	1.00		1.00		1.00	
Specificity^3^	0.25		0.75		0.75	
Accuracy^4^	0.67		0.89		0.89	
γ-irradiated						
Delivered dose (Gy)						
≤ 1.5^1^	2	4	6	0	6	0
> 1.5	0	3	0	3	0	3
Sensitivity^2^	1.00		1.00		1.00	
Specificity^3^	0.33		1.00		1.00	
Accuracy^4^	0.56		1.00		1.00	

^1^a binary category (≤ 1.5 Gy / > 1.5 Gy) to identify the subjects likely to suffer from acute radiation syndrome several days after radiation exposure (Rothkamm et al. 2013). Samples with true dose of 0 Gy was excluded in this comparison

^2^Sensitivity = true positives/(true positives + false negatives)

^3^Specificity = true negatives/(true negatives + false positives)

^4^Accuracy = (true positive + true negative)/total

**Fig. 2:**
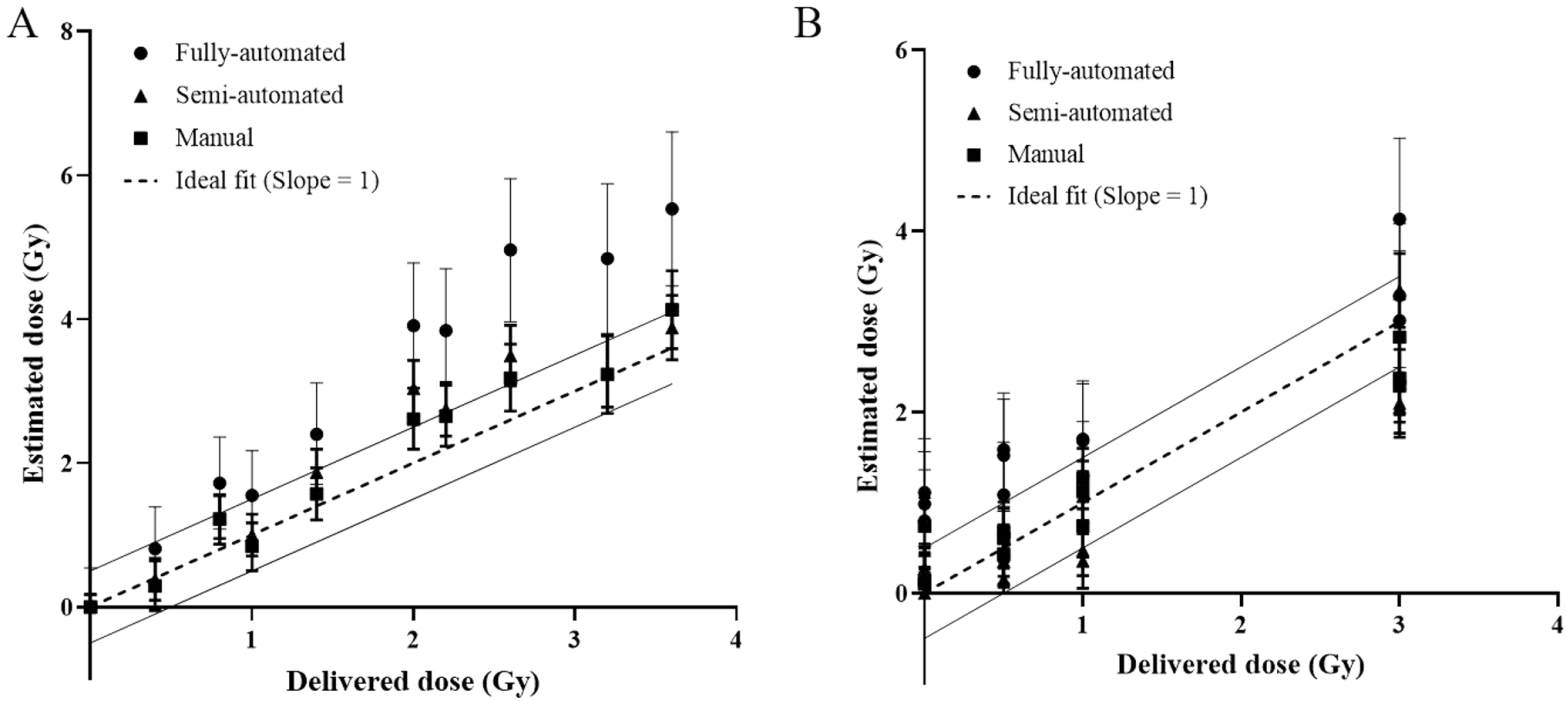
Dose prediction using fully-automated, semi-automated and manual mode with automated micronucleus scoring system. Blind samples were irradiated with X-ray **A** and γ-ray **B**. Symbols and error bars represent estimated doses and corresponding 95% confidence intervals. The dashed and solid lines represent ideal fit to estimate accurate delivered dose and their ± 0.5 Gy intervals

Next, we compared the dose estimation between the scoring modes. Of the 10 X-irradiated samples, actual doses fell within the 95% confidence interval of dose estimates for 7 and 10 samples for semi-automated and manual modes, respectively, whereas only 3 samples had accurate dose estimates in the fully-automated mode (Fig. 2A). Similar to this result, semi-automated and manual modes estimated a more accurate dose of 12 γ-irradiated samples (8 for semi-automated, 10 for manual vs. 4 for fully-automated modes; Fig. 2B). These findings indicate that a manual inspection step following automated scoring improves the accuracy of dose prediction.

To investigate the impact of sex on MN dose–response curves, our MN scoring data were divided and dose response curves for males and females were reconstructed (Table 4).

**Table 4:** Coefficients of calibration curves for micronuclei in male and female lymphocytes scored by different methods^1^

	c	*α*	*β*
Fully-automated			
Pooled	0.018 (± 0.0016)	0.024 (± 0.0039)	0.0080 (± 0.0012)
Male	0.018 (± 0.0019)	0.020 (± 0.0043)	0.0081 (± 0.0013)
Female	0.018 (± 0.0015)	0.027 (± 0.0038)	0.0079 (± 0.0012)
Semi-automated			
Pooled	0.0096 (± 0.0011)	0.017 (± 0.0031)	0.011 (± 0.0010)
Male	0.0088 (± 0.0013)	0.015 (± 0.0035)	0.011 (± 0.0011)
Female	0.010 (± 0.0012)	0.019 (± 0.0033)	0.011 (± 0.0011)
Manual			
Pooled	0.020 (± 0.0018)	0.026 (± 0.0045)	0.014 (± 0.0014)
Male	0.016 (± 0.0026)	0.027 (± 0.0066)	0.012 (± 0.0021)
Female	0.023 (± 0.0013)	0.025 (± 0.0033)	0.014 (± 0.0010)

^1^linear quadratic model was applied for dose response curves as follows: $y=c+\alpha D +\beta {D}^{2}$, where *y* is the MN frequency per BN cell, *c* is the spontaneous MN frequency, *α* is a linear component of a curve, *β* is a quadratic component of a curve, and *D* is the radiation dose

Table 5, Supplementary Tables 3 and 4 show the dose predictions using pooled and sex-specific dose response curves with different scoring modes. The use of sex-specific curves seemed to further improve dose prediction of semi-automated and manual modes, but statistical significance between the sexes was not observed.

**Table:** Comparison of dose estimation between pooled and sex-specific dose response curves

	Dose estimation accuracy^1^		Mean of absolute difference^2^	
	Pooled curve	Sex-specific curve	Pooled curve	Sex-specific curve
All				
Fully-automated	0.32	0.27	0.96	0.95
Semi-automated	0.55	0.59	0.38	0.37
Manual	0.68	0.82	0.31	0.28
X-irradiated				
Fully-automated	0.30	0.20	1.24	1.26
Semi-automated	0.50	0.50	0.40	0.41
Manual	0.60	0.80	0.34	0.31
γ-irradiated				
Fully-automated	0.33	0.33	0.73	0.70
Semi-automated	0.58	0.67	0.36	0.35
Manual	0.75	0.83	0.29	0.26

^1^Dose estimates were considered accurate, when actual doses fell within the 95% confidence interval of dose estimates

^2^Absolute difference between estimated and actual irradiated dose was calculated

## Discussion

The MN assay is a valuable tool for radiation biodosimetry that overcomes the limitations of the dicentric chromosome assay (Vral et al. 2011). Automated MN scoring using the Metafer slide-scanning system has many advantages over the conventional manual MN assay, enhancing throughput and reducing laborious and time-consuming tasks (Seager et al. 2014; Decordier et al. 2009). We found that dose estimation of the automated MN scoring can be improved by correcting automatic scoring errors.

Automated scoring tends to have a high false-positive rate (Seager et al. 2014). We evaluated the false detection rates of our automated scoring system. Only 0.72–2.20% of the scored BN cells did not comply with the standardized scoring criteria (Fenech et al. 2003); that is, most of automatically identified BN cells were correctly detected. Our false positive BN (0.72–2.20%) and MN frequency (0.67–1.50%) was comparable to that reported by Willems et al. (2010) [6.28% false positive BN rate, 1% false positive MN yields]. The error rates of BN and MN tend to increase with the radiation dose, which may be related to radiation-induced cell death, including apoptosis (Boreham et al. 2000). This reduces the accuracy of the fully-automated scoring mode.

To adjust detection errors occurring during automated micronucleus assay, a visual inspection of BN cells on the automated scoring-produced image gallery was performed. In this method, false-positive and false-negative MN scoring was corrected and false-positive BN cells were rejected. Therefore, the ability to identify individuals at risk of acute radiation syndrome in a triage and the accuracy of dose estimation were improved relative to fully-automated scoring. Similarly, the MultiBiodose study and RENEB intercomparison exercises have shown the higher accuracy of semi-automated micronucleus scoring (Depuydt et al. 2017; Thierens et al. 2014). Our study found that visual inspection following automated scoring can improve CBMN assay performance by comparing dose estimation for blind samples irradiated with 12 different doses with manual mode as well as semi-automated mode.

MN frequency can be affected by various factors such as exposure to environmental mutagens, dietary factors, age and sex (IAEA 2011). In the present study, dose estimates of 3 blind samples exposed to 0 Gy ^60^Co tended to be somewhat overestimated. The three donors (age: 35 to 50) were older than subjects for MN dose–response curve (age: 29 to 34), so donor age but also history of exposure to environmental clastogens and aneugens could be contributing factors. Various confounding factors influencing the spontaneous MN frequency assay could be a problem in real radiological accident. The discrimination of centromere-negative or positive MN could overcome the limitation because age increases mainly centromere-positive MN (Thierens et al. 1999, 2000). Indeed, it would be helpful to more precisely assess background MN frequencies in various age groups and investigate the confounding factors such as the antecedent exposure history.

Females are known to have higher spontaneous MN frequencies than males (Bonassi et al. 2001; Fenech and Bonassi 2011; Fenech et al. 1999, 1994). Female baseline MN frequencies are higher by 1.4–1.65-fold depending on age (Fenech et al. 1994), with the difference increasing with age (Bonassi et al. 2001; Fenech and Bonassi 2011). We split our automated MN scoring data based on sex. The use of sex-specific curves seemed to further improve the dose prediction of semi-automated and manual modes, but we could not see a statistical significance. Our subjects for MN dose response curve consisted of 3 males and 3 females so the small numbers might be not be sufficient for statistical significance. Larger studies are needed to confirm the improvement of dose estimation by the use of sex-specific curves.

To determine the best way to use automated scoring, we extensively compared its characteristics with those of other scoring methods. Visual inspection improved accuracy, but the additional steps required increase of scoring time. Approximately 10 min for fully-automated mode, 15 min for semi-automated mode, and 30 min for manual mode was required to scan and analyze one slide. The best choice of scoring systems would therefore depend on the purpose. When the main goal of the MN assay is to identify subjects who need urgent clinical treatment for a triage, more rapid method would be preferred. But if more precision is required, scoring methods with visual inspection, semi-automated or manual, should be chosen over fully-automated scoring. Considering that the same images can be used for both automated and visually inspected methods, those performing the assay have significant technical and temporal latitude to adjust the assay to achieve the accuracy required for specific situations. Additional visual inspection following automated scoring can be the best approach. In addition, the use of sex-specific curves can be considered as a simple way to further improve dose estimation.

In addition, the energy of the photon radiation source could affect the MN frequency induced by radiation. Our dose–response curve was constructed using blood samples exposed to γ-rays from ^60^Co with a mean energy of 1.2 MeV. The dose of γ-irradiated blind samples could be estimated with higher accuracy and specificity than that of the samples exposed to 250 kVp X rays. This might be explained by the higher relative biological effectiveness (RBE) of soft vs hard photons (Schmid et al. 2002). The dependence of RBE on the energy of sparsely ionizing radiations has been attributed to microdosimetric differences between these radiations. Lloyd et al. (1975) and Schmid et al. (2002) showed 250 kV X rays produced higher α coefficient than ^60^Co γ rays. These differences might cause the overestimation of exposed dose in X-irradiated samples when using dose–response curve generated using ^60^Co γ rays. Additional generation of dose–response curves for X-irradiation of appropriate energy could improve the accuracy of dose estimation.

Our study provides strong evidence showing that visual inspection of images captured by an automated MN system is necessary for accurate dosimetry. Using a validation data set of 22 blind samples, we found that the correction of automated scoring improved the performance of automated MN scoring. Our findings could be useful for performing radiation dosimetry on large numbers of people rapidly, accurately, and efficiently.

## Supplementary Information

Below is the link to the electronic supplementary material.

Supplementary file1 (DOCX 47 KB)

## Data Availability

The datasets generated during and/or analysed during the current study are available from the corresponding author on reasonable request.

## References

[CR1] Ainsbury EA, Lloyd DC (2010). Dose estimation software for radiation biodosimetry. Health Phys.

[CR2] Bonassi S, Fenech M, Lando C, Lin YP, Ceppi M, Chang WP, Holland N, Kirsch-Volders M, Zeiger E, Ban S, Barale R, Bigatti MP, Bolognesi C, Jia C, Di Giorgio M, Ferguson LR, Fucic A, Lima OG, Hrelia P, Krishnaja AP, Lee TK, Migliore L, Mikhalevich L, Mirkova E, Mosesso P, Muller WU, Odagiri Y, Scarffi MR, Szabova E, Vorobtsova I, Vral A, Zijno A (2001). HUman MicroNucleus project: international database comparison for results with the cytokinesis-block micronucleus assay in human lymphocytes: I Effect of laboratory protocol, scoring criteria, and host factors on the frequency of micronuclei. Environm Mol Mutag.

[CR3] Boreham DR, Dolling JA, Maves SR, Siwarungsun N, Mitchel RE (2000). Dose-rate effects for apoptosis and micronucleus formation in gamma-irradiated human lymphocytes. Radiat Res.

[CR4] Chung HW, Ryu EK, Kim YJ, Ha SW (1996). Chromosome aberrations in workers of nuclear-power plants. Mutat Res.

[CR5] Decordier I, Papine A, Plas G, Roesems S, Vande Loock K, Moreno-Palomo J, Cemeli E, Anderson D, Fucic A, Marcos R, Soussaline F, Kirsch-Volders M (2009). Automated image analysis of cytokinesis-blocked micronuclei: an adapted protocol and a validated scoring procedure for biomonitoring. Mutagenesis.

[CR6] Depuydt J, Baeyens A, Barnard S, Beinke C, Benedek A, Beukes P, Buraczewska I, Darroudi F, De Sanctis S, Dominguez I, Monteiro Gil O, Hadjidekova V, Kis E, Kulka U, Lista F, Lumniczky K, M'Kacher R, Moquet J, Obreja D, Oestreicher U, Pajic J, Pastor N, Popova L, Regalbuto E, Ricoul M, Sabatier L, Slabbert J, Sommer S, Testa A, Thierens H, Wojcik A, Vral A (2017). RENEB intercomparison exercises analyzing micronuclei (Cytokinesis-block Micronucleus Assay). Int J Radiat Biol.

[CR7] Fenech M, Bonassi S (2011). The effect of age, gender, diet and lifestyle on DNA damage measured using micronucleus frequency in human peripheral blood lymphocytes. Mutagenesis.

[CR8] Fenech M, Morley AA (1985). Measurement of micronuclei in lymphocytes. Mutat Res.

[CR9] Fenech M, Neville S, Rinaldi J (1994). Sex is an important variable affecting spontaneous micronucleus frequency in cytokinesis-blocked lymphocytes. Mutat Res.

[CR10] Fenech M, Holland N, Chang WP, Zeiger E, Bonassi S (1999). The HUman MicroNucleus Project–An international collaborative study on the use of the micronucleus technique for measuring DNA damage in humans. Mutat Res.

[CR11] Fenech M, Chang WP, Kirsch-Volders M, Holland N, Bonassi S, Zeiger E (2003). HUMN project: detailed description of the scoring criteria for the cytokinesis-block micronucleus assay using isolated human lymphocyte cultures. Mutat Res.

[CR12] IAEA (2011). Cytogenetic dosimetry: applications in preparedness for and response to radiation emergencies.

[CR13] ISO (2014) Radiological protection — Performance criteria for laboratories using the cytokinesis block micronucleus (CBMN) assay in peripheral blood lymphocytes for biological dosimetry. ISO 17099. ISO, Geneva

[CR14] Lloyd DC, Purrott RJ, Dolphin GW, Bolton D, Edwards AA, Corp MJ (1975). The relationship between chromosome aberrations and low LET radiation dose to human lymphocytes. Int J Radiat Biol Relat Stud Phys Chem Med.

[CR15] Lue SW, Repin M, Mahnke R, Brenner DJ (2015). Development of a High-Throughput and Miniaturized Cytokinesis-Block Micronucleus Assay for Use as a Biological Dosimetry Population Triage Tool. Radiat Res.

[CR16] OECD (2016) Test No 487 In Vitro Mammalian Cell Micronucleus Test. doi:10.1787/9789264264861-en

[CR17] Ramalho AT, Nascimento AC (1991). The fate of chromosomal aberrations in 137Cs-exposed individuals in the Goiania radiation accident. Health Phys.

[CR18] Romm H, Barnard S, Boulay-Greene H, De Amicis A, De Sanctis S, Franco M, Herodin F, Jones A, Kulka U, Lista F, Martigne P, Moquet J, Oestreicher U, Rothkamm K, Thierens H, Valente M, Vandersickel V, Vral A, Braselmann H, Meineke V, Abend M, Beinke C (2013). Laboratory intercomparison of the cytokinesis-block micronucleus assay. Radiat Res.

[CR19] Rothkamm K, Beinke C, Romm H, Badie C, Balagurunathan Y, Barnard S, Bernard N, Boulay-Greene H, Brengues M, De Amicis A, De Sanctis S, Greither R, Herodin F, Jones A, Kabacik S, Knie T, Kulka U, Lista F, Martigne P, Missel A, Moquet J, Oestreicher U, Peinnequin A, Poyot T, Roessler U, Scherthan H, Terbrueggen B, Thierens H, Valente M, Vral A, Zenhausern F, Meineke V, Braselmann H, Abend M (2013). Comparison of established and emerging biodosimetry assays. Radiat Res.

[CR20] Schmid E, Regulla D, Kramer HM, Harder D (2002). The effect of 29 kV X rays on the dose response of chromosome aberrations in human lymphocytes. Radiat Res.

[CR21] Seager AL, Shah UK, Brusehafer K, Wills J, Manshian B, Chapman KE, Thomas AD, Scott AD, Doherty AT, Doak SH, Johnson GE, Jenkins GJ (2014). Recommendations, evaluation and validation of a semi-automated, fluorescent-based scoring protocol for micronucleus testing in human cells. Mutagenesis.

[CR22] Shibai-Ogata A, Kakinuma C, Hioki T, Kasahara T (2011). Evaluation of high-throughput screening for in vitro micronucleus test using fluorescence-based cell imaging. Mutagenesis.

[CR23] Slozina N, Neronova E, Nikiforov A (2001). Persistence of dicentrics in Chernobyl clean-up workers who suffered from low doses of radiation. Appl Radiat Isot.

[CR24] Suto Y, Hirai M, Akiyama M, Kobashi G, Itokawa M, Akashi M, Sugiura N (2013). Biodosimetry of restoration workers for the Tokyo Electric Power Company (TEPCO) Fukushima Daiichi nuclear power station accident. Health Phys.

[CR25] Thierens H, Vral A, Barbe M, Aousalah B, De Ridder L (1999). A cytogenetic study of nuclear power plant workers using the micronucleus-centromere assay. Mutat Res.

[CR26] Thierens H, Vral A, Morthier R, Aousalah B, De Ridder L (2000). Cytogenetic monitoring of hospital workers occupationally exposed to ionizing radiation using the micronucleus centromere assay. Mutagenesis.

[CR27] Thierens H, Vral A, Vandevoorde C, Vandersickel V, de Gelder V, Romm H, Oestreicher U, Rothkamm K, Barnard S, Ainsbury E, Sommer S, Beinke C, Wojcik A (2014). Is a semi-automated approach indicated in the application of the automated micronucleus assay for triage purposes?. Radiat Prot Dosimetry.

[CR28] Vral A, Verhaegen F, Thierens H, De Ridder L (1994). Micronuclei induced by fast neutrons versus 60Co gamma-rays in human peripheral blood lymphocytes. Int J Radiat Biol.

[CR29] Vral A, Fenech M, Thierens H (2011). The micronucleus assay as a biological dosimeter of in vivo ionising radiation exposure. Mutagenesis.

[CR30] Willems P, August L, Slabbert J, Romm H, Oestreicher U, Thierens H, Vral A (2010). Automated micronucleus (MN) scoring for population triage in case of large scale radiation events. Int J Radiat Biol.

