# Supplementary material for: Improving radiation dosimetry with an automated micronucleus scoring system: correction of automated scoring errors
Source: Radiat Environ Biophys. 2023 May 17;62(3):349–56. doi: 10.1007/s00411-023-01030-7 (PMC10356889; doi:10.1007/s00411-023-01030-7)
Supplement: Supplementary file 1 — Supplementary file1 (DOCX 47 KB) [file 411_2023_1030_MOESM1_ESM.docx]

Supplementary Table 1. Micronuclei frequencies and distributions in human lymphocytes by semi-automated scoring

| Dose (Gy) | No. of BN | No. of MN | Distribution of MN | | | | | | | | | Dispersion index (${\sigma^{2}}/{y)}$ | MN  frequency | | |
| --- | --- | --- | --- | --- | --- | --- | --- | --- | --- | --- | --- | --- | --- | --- | --- |
|  |  |  | 0 | 1 | 2 | | | 3 | 4 | | |  |  |  |  |
| 0 | 15880 | 186 | 15706 | 163 | | 10 | 1 | | | 0 | 1.13 | | | 0.012 |  |
| 0.1 | 17847 | 177 | 17676 | 165 | | 6 | 0 | | | 0 | 1.06 | | | 0.010 |  |
| 0.25 | 17851 | 261 | 17602 | 237 | | 12 | 0 | | | 0 | 1.08 | | | 0.015 |  |
| 0.5 | 17778 | 358 | 17440 | 319 | | 18 | 1 | | | 0 | 1.1 | | | 0.020 |  |
| 0.75 | 17829 | 479 | 17371 | 439 | | 18 | 0 | | | 1 | 1.07 | | | 0.027 |  |
| 1.0 | 17779 | 650 | 17167 | 576 | | 34 | 2 | | | 0 | 1.09 | | | 0.037 |  |
| 2.0 | 17764 | 1681 | 16256 | 1358 | | 132 | 13 | | | 5 | 1.14 | | | 0.095 |  |
| 3.0 | 17297 | 2843 | 14933 | 1947 | | 358 | 56 | | | 3 | 1.22 | | | 0.164 |  |
| 4.0 | 17734 | 4394 | 14295 | 2640 | | 656 | 130 | | | 13 | 1.26 | | | 0.248 |  |

Supplementary Table 2. Micronuclei frequencies and distributions in human lymphocytes by manual scoring

| Dose (Gy) | No. of BN | No. of MN | Distribution of MN | | | | | | | | | Dispersion index (${\sigma^{2}}/{y)}$ | MN  frequency | | |
| --- | --- | --- | --- | --- | --- | --- | --- | --- | --- | --- | --- | --- | --- | --- | --- |
|  |  |  | 0 | 1 | 2 | | | 3 | 4 | | |  |  |  |  |
| 0 | 15863 | 356 | 15525 | 321 | | 16 | 1 | | | 0 | 1.08 | | | 0.022 |  |
| 0.1 | 17870 | 358 | 17525 | 332 | | 13 | 0 | | | 0 | 1.05 | | | 0.020 |  |
| 0.25 | 17856 | 491 | 17388 | 446 | | 21 | 1 | | | 0 | 1.07 | | | 0.027 |  |
| 0.5 | 17791 | 663 | 17171 | 579 | | 39 | 2 | | | 0 | 1.1 | | | 0.037 |  |
| 0.75 | 17804 | 769 | 17066 | 709 | | 28 | 0 | | | 1 | 1.05 | | | 0.043 |  |
| 1.0 | 17816 | 994 | 16874 | 893 | | 46 | 3 | | | 0 | 1.05 | | | 0.056 |  |
| 2.0 | 17843 | 2387 | 15713 | 1903 | | 202 | 20 | | | 5 | 1.11 | | | 0.134 |  |
| 3.0 | 17330 | 3870 | 14085 | 2693 | | 484 | 63 | | | 5 | 1.14 | | | 0.223 |  |
| 4.0 | 17604 | 5823 | 13006 | 3561 | | 866 | 154 | | | 17 | 1.16 | | | 0.331 |  |

Supplementary Table 3. Dose estimation results of X-irradiated samples according to scoring methods

| Sample ID | | Sex | MN frequency | | | Dispersion index (${\sigma^{2}}/{y)}$ | Delivered dose (Gy) | General dose response curve | | | | | | Sex specific dose response curve | | | | | |  |
| --- | --- | --- | --- | --- | --- | --- | --- | --- | --- | --- | --- | --- | --- | --- | --- | --- | --- | --- | --- | --- |
|  |  |  |  |  |  |  |  | Dose | | 95% LL | | 95% UL | | Dose | | 95% LL | | 95% UL | |  |
|  | | | |  | Fully-automated | | | |  | |  | |  | |  | |  | |  | |
| X01 | | F | 0.121 | | | 1.04 | 1.40 | 2.40 | | 1.70 | | 3.11 | | 2.29 | | 1.54 | | 3.04 | |  |
| X02 | | F | 0.320 | | | 0.95 | 3.20 | 4.84 | | 3.80 | | 5.88 | | 4.70 | | 3.62 | | 5.78 | |  |
| X03 | | M | 0.042 | | | 1.07 | 0.40 | 0.81 | | 0.23 | | 1.39 | | 0.89 | | 0.31 | | 1.47 | |  |
| X04 | | F | 0.332 | | | 0.97 | 2.60 | 4.96 | | 3.96 | | 5.95 | | 4.82 | | 3.78 | | 5.86 | |  |
| X05 | | M | 0.233 | | | 0.97 | 2.00 | 3.91 | | 3.04 | | 4.78 | | 4.05 | | 3.15 | | 4.95 | |  |
| X06 | | M | 0.016 | | | 1.07 | 0.00 | 0.00 | | 0.00 | | 0.54 | | 0.00 | | 0.00 | | 0.54 | |  |
| X07 | | M | 0.074 | | | 1.01 | 1.00 | 1.55 | | 0.92 | | 2.17 | | 1.66 | | 1.03 | | 2.29 | |  |
| X08 | | M | 0.394 | | | 0.97 | 3.60 | 5.53 | | 4.46 | | 6.60 | | 5.68 | | 4.56 | | 6.80 | |  |
| X09 | | F | 0.082 | | | 1.11 | 0.80 | 1.72 | | 1.08 | | 2.36 | | 1.61 | | 0.93 | | 2.30 | |  |
| X10 | | M | 0.226 | | | 0.94 | 2.20 | 3.84 | | 2.73 | | 4.70 | | 3.98 | | 3.09 | | 4.87 | |  |
|  | |  |  | | |  |  |  | |  | |  | |  | |  | |  | |  |
|  | | | |  | Semi-automated | | | |  | |  | |  | |  | |  | |  | |
| X01 | | F | 0.080 | | | 1.09 | 1.40 | 1.87 | | 1.55 | | 2.19 | | 1.79 | | 1.45 | | 2.13 | |  |
| X02 | | F | 0.184 | | | 1.06 | 3.20 | 3.27 | | 2.78 | | 3.77 | | 3.17 | | 2.66 | | 3.68 | |  |
| X03 | | M | 0.018 | | | 0.98 | 0.40 | 0.38 | | 0.09 | | 0.67 | | 0.44 | | 0.13 | | 0.76 | |  |
| X04 | | F | 0.204 | | | 1.09 | 2.60 | 3.49 | | 3.07 | | 3.91 | | 3.39 | | 2.94 | | 3.83 | |  |
| X05 | | M | 0.163 | | | 1.08 | 2.00 | 3.04 | | 2.64 | | 3.43 | | 3.14 | | 2.70 | | 3.58 | |  |
| X06 | | M | 0.006 | | | 0.99 | 0.00 | 0.00 | | 0.00 | | 0.17 | | 0.00 | | 0.00 | | 0.26 | |  |
| X07 | | M | 0.038 | | | 1.05 | 1.00 | 1.00 | | 0.71 | | 1.29 | | 1.07 | | 0.76 | | 1.39 | |  |
| X08 | | M | 0.243 | | | 1.22 | 3.60 | 3.88 | | 3.43 | | 4.33 | | 4.00 | | 3.50 | | 4.50 | |  |
| X09 | | F | 0.048 | | | 1.20 | 0.80 | 1.25 | | 0.95 | | 1.54 | | 1.18 | | 0.87 | | 1.49 | |  |
| X10 | | M | 0.140 | | | 1.08 | 2.20 | 2.75 | | 2.37 | | 3.12 | | 2.85 | | 2.43 | | 3.26 | |  |
|  | |  |  | | |  |  |  | |  | |  | |  | |  | |  | |  |
|  |  | | | Manual | |  |  |  | | | | | |  | | | | | |  |
| X01 | | F | 0.094 | | | 0.98 | 1.40 | 1.57 | | 1.21 | | 1.93 | | 1.50 | | 1.24 | | 1.76 | |  |
| X02 | | F | 0.244 | | | 0.93 | 3.20 | 3.23 | | 2.69 | | 3.77 | | 3.14 | | 2.73 | | 3.55 | |  |
| X03 | | M | 0.028 | | | 1.00 | 0.40 | 0.29 | | 0.00 | | 0.64 | | 0.40 | | 0.00 | | 0.92 | |  |
| X04 | | F | 0.239 | | | 1.04 | 2.60 | 3.18 | | 2.72 | | 3.65 | | 3.09 | | 2.77 | | 3.41 | |  |
| X05 | | M | 0.180 | | | 1.04 | 2.00 | 2.61 | | 2.19 | | 3.04 | | 2.70 | | 2.00 | | 3.41 | |  |
| X06 | | M | 0.013 | | | 0.99 | 0.00 | 0.00 | | 0.00 | | 0.17 | | 0.00 | | 0.00 | | 0.49 | |  |
| X07 | | M | 0.051 | | | 1.00 | 1.00 | 0.84 | | 0.50 | | 1.17 | | 0.91 | | 0.38 | | 1.45 | |  |
| X08 | | M | 0.357 | | | 1.08 | 3.60 | 4.13 | | 3.59 | | 4.67 | | 4.27 | | 3.36 | | 5.18 | |  |
| X09 | | F | 0.071 | | | 1.12 | 0.80 | 1.22 | | 0.87 | | 1.56 | | 1.15 | | 0.89 | | 1.40 | |  |
| X10 | | M | 0.183 | | | 1.04 | 2.20 | 2.65 | | 2.23 | | 3.08 | | 2.74 | | 2.03 | | 3.45 | |  |

When actual delivered dose fell within 95% confidence interval of a dose estimate, we considered it is correctly estimated.

Supplementary Table 4. Dose estimation results of γ-irradiated samples according to scoring methods

| Sample ID | | Sex | | MN frequency | | | Dispersion index (${\sigma^{2}}/{y)}$ | Delivered dose (Gy) | General dose response curve | | | Sex specific dose response curve | | |
| --- | --- | --- | --- | --- | --- | --- | --- | --- | --- | --- | --- | --- | --- | --- |
|  |  |  |  |  |  |  |  |  | Dose | 95% LL | 95% UL | Dose | 95% LL | 95% UL |
|  | | |  | | | Fully-automated | | |  |  |  |  |  |  |
| G01 | | M | | 0.053 | | | 1.24 | 0.5 | 1.09 | 0.50 | 1.67 | 1.18 | 0.59 | 1.76 |
| G02 | | F | | 0.080 | | | 1.16 | 1.0 | 1.68 | 1.06 | 2.31 | 1.58 | 0.91 | 2.26 |
| G03 | | M | | 0.081 | | | 1.33 | 1.0 | 1.70 | 1.06 | 2.35 | 1.82 | 1.17 | 2.46 |
| G04 | | F | | 0.049 | | | 1.21 | 0.0 | 0.99 | 0.40 | 1.57 | 0.92 | 0.28 | 1.55 |
| G05 | | F | | 0.076 | | | 1.36 | 0.5 | 1.59 | 0.96 | 2.21 | 1.49 | 0.81 | 2.17 |
| G06 | | F | | 0.042 | | | 1.21 | 0.0 | 0.80 | 0.23 | 1.37 | 0.74 | 0.12 | 1.36 |
| G07 | | F | | 0.182 | | | 1.11 | 3.0 | 3.29 | 2.49 | 4.08 | 3.16 | 2.32 | 4.00 |
| G08 | | F | | 0.256 | | | 1.02 | 3.0 | 4.13 | 3.24 | 5.03 | 4.03 | 3.09 | 4.98 |
| G09 | | F | | 0.062 | | | 1.17 | 1.0 | 1.29 | 0.69 | 1.90 | 1.21 | 0.56 | 1.86 |
| G10 | | F | | 0.072 | | | 1.15 | 0.5 | 1.52 | 0.90 | 2.14 | 1.43 | 0.76 | 2.10 |
| G11 | | M | | 0.054 | | | 1.29 | 0.0 | 1.11 | 0.52 | 1.71 | 1.20 | 0.61 | 1.80 |
| G12 | | M | | 0.162 | | | 1.08 | 3.0 | 3.01 | 2.24 | 3.78 | 3.15 | 2.36 | 3.94 |
|  | |  | |  | | |  |  |  |  |  |  |  |  |
|  | | |  | | | Semi-automated | | |  |  |  |  |  |  |
| G01 | | M | | 0.012 | | | 1.14 | 0.5 | 0.15 | -0.14 | 0.44 | 0.21 | -0.11 | 0.52 |
| G02 | | F | | 0.041 | | | 1.14 | 1.0 | 1.07 | 0.80 | 1.35 | 1.00 | 0.71 | 1.29 |
| G03 | | M | | 0.017 | | | 0.98 | 1.0 | 0.36 | 0.05 | 0.66 | 0.42 | 0.09 | 0.74 |
| G04 | | F | | 0.009 | | | 1.10 | 0.0 | 0.00 | 0.00 | 0.29 | 0.00 | 0.00 | 0.27 |
| G05 | | F | | 0.017 | | | 1.09 | 0.5 | 0.35 | 0.07 | 0.64 | 0.30 | 0.00 | 0.61 |
| G06 | | F | | 0.015 | | | 1.06 | 0.0 | 0.27 | 0.00 | 0.54 | 0.22 | -0.07 | 0.51 |
| G07 | | F | | 0.094 | | | 1.13 | 3.0 | 2.10 | 1.77 | 2.43 | 2.01 | 1.66 | 2.36 |
| G08 | | F | | 0.191 | | | 1.14 | 3.0 | 3.35 | 2.94 | 3.75 | 3.24 | 2.81 | 3.67 |
| G09 | | F | | 0.020 | | | 1.29 | 1.0 | 0.47 | 0.19 | 0.75 | 0.42 | 0.12 | 0.71 |
| G10 | | F | | 0.020 | | | 1.21 | 0.5 | 0.47 | 0.19 | 0.75 | 0.41 | 0.12 | 0.71 |
| G11 | | M | | 0.008 | | | 1.07 | 0.0 | 0.00 | 0.00 | 0.27 | 0.00 | 0.00 | 0.35 |
| G12 | | M | | 0.091 | | | 1.26 | 3.0 | 2.06 | 1.72 | 2.39 | 2.15 | 1.78 | 2.52 |
|  | |  | |  | | |  |  |  |  |  |  |  |  |
|  |  | | | | Manual | |  |  |  | | |  | | |
| G01 | | M | | 0.033 | | | 1.11 | 0.5 | 0.43 | 0.10 | 0.75 | 0.52 | 0.01 | 1.04 |
| G02 | | F | | 0.074 | | | 1.07 | 1.0 | 1.27 | 0.93 | 1.60 | 1.20 | 0.96 | 1.44 |
| G03 | | M | | 0.046 | | | 1.00 | 1.0 | 0.74 | 0.40 | 1.08 | 0.82 | 0.29 | 1.35 |
| G04 | | F | | 0.022 | | | 1.16 | 0.0 | 0.10 | 0.00 | 0.45 | 0.00 | 0.00 | 0.26 |
| G05 | | F | | 0.040 | | | 1.07 | 0.5 | 0.60 | 0.27 | 0.94 | 0.52 | 0.27 | 0.77 |
| G06 | | F | | 0.046 | | | 0.99 | 0.0 | 0.74 | 0.43 | 1.06 | 0.66 | 0.44 | 0.89 |
| G07 | | F | | 0.158 | | | 1.05 | 3.0 | 2.38 | 1.97 | 2.78 | 2.30 | 2.02 | 2.58 |
| G08 | | F | | 0.201 | | | 1.15 | 3.0 | 2.83 | 2.39 | 3.26 | 2.74 | 2.44 | 3.04 |
| G09 | | F | | 0.066 | | | 1.35 | 1.0 | 1.12 | 0.79 | 1.46 | 1.05 | 0.81 | 1.30 |
| G10 | | F | | 0.044 | | | 1.00 | 0.5 | 0.68 | 0.35 | 1.01 | 0.60 | 0.35 | 0.85 |
| G11 | | M | | 0.024 | | | 1.17 | 0.0 | 0.14 | 0.00 | 0.50 | 0.26 | 0.00 | 0.79 |
| G12 | | M | | 0.150 | | | 1.11 | 3.0 | 2.29 | 1.89 | 2.69 | 2.37 | 1.71 | 3.04 |

When actual delivered dose fell within 95% confidence interval of a dose estimate, we considered it is correctly estimated.
